# Cardiovascular Effects, Antioxidant Activity, and Phytochemical Analysis of *Rubus ulmifolius* Schott Leaves

**DOI:** 10.3390/plants14162513

**Published:** 2025-08-12

**Authors:** Afaf Mehiou, Chaimae Alla, Zachée Louis Evariste Akissi, Ikram Dib, Sanae Abid, Ali Berraaouan, Hassane Mekhfi, Abdelkhaleq Legssyer, Abderrahim Ziyyat, Sevser Sahpaz

**Affiliations:** 1 Laboratory of Bioresources, Biotechnologies, Ethnopharmacology and Health, Department of Biology, Faculty of Sciences, University Mohammed First, Oujda 60000, Morocco; afaf.mehiou@ump.ac.ma (A.M.); chaimae.alla@ump.ac.ma (C.A.); abid.sanae24@ump.ac.ma (S.A.); a.berraaouan@ump.ac.ma (A.B.); h.mekhfi@ump.ac.ma (H.M.); a.legssyer@ump.ac.ma (A.L.); ar.ziyyat@ump.ac.ma (A.Z.); 2 BioEcoAgro Joint Cross-Border Research Unit 1158, University of Lille, INRAE, University of Liège, UPJV, JUNIA, University of Artois, University of Littoral Côte d’Opale, Cité Scientifique, 59655 Villeneuve d’Ascq, France; zachee.akissi@univ-lille.fr; 3 Higher Institute of Nursing Professions and Health Techniques (ISPITS), Hassani Hospital, Nador 62000, Morocco; i.dib@ump.ac.ma

**Keywords:** wild blackberry, blood pressure, vasodilation, hypertension, antioxidant, polyphenols, tannins, functional food

## Abstract

Wild blackberry (*Rubus ulmifolius* Schott) is a culinary and medicinal plant traditionally used to treat various ailments, including hypertension. This study evaluated the vasorelaxant effects of five crude leaf extracts of *R. ulmifolius* (hexane, dichloromethane, ethyl acetate, methanol, and aqueous), as well as the hypotensive and antioxidant activities of its methanolic extract (MERu), and analyzed its phytochemical profile. Crude extracts, obtained using a Soxhlet apparatus, were tested in vitro on isolated rat aortic rings precontracted with phenylephrine. The hypotensive effect of MERu was examined in vivo in normotensive rats, and its antioxidant activity was assessed using the DPPH assay. Total phenolic and tannin contents were quantified by the Folin–Ciocalteu and hide powder methods, respectively, while UHPLC-MS was used to identify its phytochemicals. All crude extracts induced concentration-dependent vasorelaxation, with MERu showing the strongest effect (59.31% relaxation at 10^−1^ g/L). Intravenous MERu induced significant blood pressure reductions in rats, starting at 1 mg/kg. At 20 mg/kg, systolic, diastolic, and mean arterial pressures dropped by 38.61%, 51.58%, and 45.19%, respectively. MERu also demonstrated potent antioxidant activity and was rich in polyphenols, particularly tannins. Sixteen compounds were identified, notably rubanthrone A, a galloyl-bis-HHDP glucose derivative, ellagic acid, and quercetin-3-O-β-D-glucuronide. These results suggest that *R. ulmifolius* may have therapeutic potential for hypertension and exhibits promising characteristics as a functional food.

## 1. Introduction

Hypertension is a major risk factor for cardiovascular diseases, accounting for 47% of ischemic heart disease cases and 54% of strokes, making it one of the leading causes of mortality worldwide [1]. This condition is defined by a persistent elevation of systolic blood pressure (SBP) at or above 140 mmHg and/or diastolic blood pressure (DBP) at or above 90 mmHg [2]. More than 90% of hypertensive individuals suffer from primary or essential hypertension, the causes of which remain unknown. In comparison, the remaining 10% are affected by secondary hypertension linked to other treatable conditions such as kidney or thyroid diseases [3]. Several factors contribute to the development of hypertension. Non-modifiable risk factors include age, male sex, premature menopause, and heredity. In contrast, modifiable risk factors are largely associated with lifestyle. These include unfavorable socioeconomic conditions, stress, physical inactivity, smoking, obesity, excessive sodium and alcohol intake, and an unbalanced diet [1,4]. In 2000, approximately 25% of the global population was affected by this condition. Projections indicated that this prevalence would increase by 60% by 2025, reaching 1.56 billion people [5]. The burden of hypertension is particularly significant in Africa, where nearly 50% of individuals aged 25 and older (over 150 million people) are affected. In 2019, hypertension was the leading risk factor for mortality in North Africa, accounting for 26% of deaths, while in sub-Saharan Africa, it ranked fourth among risk factors, contributing to 9% of deaths [6]. The prevention and management of hypertension primarily rely on lifestyle modifications, which also enhance the efficacy of pharmacological treatments. These interventions include adopting a healthy and balanced diet, reducing sodium and alcohol intake, increasing the consumption of plant-based foods, engaging in regular physical activity, achieving weight reduction, effectively managing stress, and quitting smoking [7]. Pharmacological treatment involves the use of various drug classes, including calcium channel blockers, angiotensin-converting enzyme inhibitors, angiotensin receptor blockers, diuretics, and beta-blockers [8].

Alongside pharmacological approaches, there is growing global interest in using medicinal plants to manage hypertension. Many individuals rely on these plants to treat various ailments, including hypertension, driving increased scientific interest in their potential therapeutic benefits [9].

*Rubus* species, belonging to the Rosaceae family and including blackberries and raspberries, have been utilized as a source of food and in traditional medicine across various cultures for centuries. Previous research has established that leaves, roots, and fruits have astringent, anti-inflammatory, and antimicrobial effects, and have been utilized to treat digestive issues, skin wounds, respiratory problems, and menstrual disorders. Additionally, *Rubus* species are rich in antioxidants, contributing to their use in promoting overall health and combating oxidative stress. *Rubus ulmifolius*, commonly known as wild blackberry, is a perennial shrub widely distributed across Europe, Asia, and North Africa [10]. It is valued not only for its medicinal properties but also for its culinary uses. Its nutrient-rich fruits are consumed fresh or processed into jams and juices [11]. The leaves are used as natural food additives, particularly as colorants [12]. In Moroccan traditional medicine, leaf powder is applied topically to treat burns [13]. Leaf macerations are used to relieve gastrointestinal disorders and diarrhea [14], while infusions are employed to alleviate menstrual pain [15] and cardiac arrhythmia [16]. Leaf decoctions are used to treat oral infections and as facial rinses to firm the skin [17]. Additionally, decoctions combined with *Cynara cardunculus* L., *Marrubium vulgare* L., *Lavandula dentata* L., and eucalyptus are traditionally used to treat headaches [17]. The leaves are also used in the management of diabetes [12]. In Algerian traditional medicine, leaf infusions are administered to treat hypertension [18] and diarrhea [19] and are also valued for their anti-inflammatory properties [20]. Furthermore, they are applied as poultices to treat skin disorders [21].

From a phytochemical perspective, *R. ulmifolius* leaves are a significant source of secondary metabolites, including flavonoids such as catechin, hyperoside, and kaempferol 3-O-rutinoside; phenolic acids such as 3-O-caffeoylquinic acid and 3-*p*-coumaroylquinic acid; anthrones such as rubanthrone A; and tannins such as ellagic acid, methyl gallate, and the glucose derivative galloyl-bis-HHDP [22,23,24,25]. Previous pharmacological studies have highlighted various biological activities of *R. ulmifolius* leaves, notably their antibacterial [23], antiviral [26], antifungal [27], antiproliferative [28], and antioxidant properties [24]. More recently, we evaluated the hypotensive and vasodilatory activities of the aqueous extract, both in vivo and ex vivo, in rats. The results were promising, particularly in terms of blood pressure reduction [25]. Although the aqueous extract exhibited a notable cardiovascular effect, it likely represents only a fraction of the plant’s phytochemical content. To broaden the range of extracted compounds and further investigate the cardiovascular effects of *R. ulmifolius*, we employed the Soxhlet extraction technique using solvents of increasing polarity, resulting in five successive crude extracts (hexane, dichloromethane, ethyl acetate, methanol, and aqueous). Regarding the toxicity of the leaf extracts, an acute toxicity study was previously conducted using the methanolic extract obtained by maceration of the aerial parts of *Rubus ulmifolius*, administered to mice at various doses (0.01, 0.1, 1, 2, 4, and 6 g/kg). The results indicated that the extract was non-toxic up to a dose of 6 g/kg body weight [29]. Similarly, administration of the methanolic extract of *R. ulmifolius*’s aerial parts at a dose of 2.5 g/kg body weight did not cause any mortality or behavioral changes in mice [30].

This study aims to evaluate the vasorelaxant effects of crude extracts of varying polarity, obtained from *R. ulmifolius* using a Soxhlet apparatus, on isolated rat aorta. Additionally, it seeks to investigate the hypotensive effect of the most active extract, the methanolic extract, assess its antioxidant activity, and determine its phytochemical composition.

## 2. Materials and Methods

### 2.1. Plant Material

The leaves of *Rubus ulmifolius* Schott (wild blackberry) were collected in September 2023 in Berkane (34°53′25.0″ N, 2°20′44.8″ W), located in the Oriental region of Morocco. The plant was identified by Professor Mostafa Elachouri, and a voucher specimen was deposited in the herbarium of the Faculty of Sciences at Mohamed First University (Oujda, Morocco) under the accession number HUMPOM741.

### 2.2. Crude Extracts Preparation

The leaves of *R. ulmifolius* were dried in the shade and ground into a fine powder. A total of 170 g of the powder was sequentially extracted using a Soxhlet apparatus with solvents of increasing polarity: hexane, dichloromethane, ethyl acetate, methanol, and distilled water. For each extraction, 600 mL of solvent was used. The extraction process was carried out over several cycles and continued until the solvent became colorless. The extraction time for each solvent was approximately 8 h. The resulting extracts were evaporated under reduced pressure at 40 °C using a rotary evaporator and subsequently stored at −20 °C until further use. These extracts are referred to as crude extracts. The extraction yields were calculated using the following formula:

Extraction yield (%) = (Weight of obtained extract [g]/Weight of dried plant material [g]) × 100

### 2.3. Experimental Animals

The experiments were conducted on male Wistar rats raised under standard breeding conditions in the animal house of the Department of Biology at the Faculty of Sciences, Mohammed First University, Oujda, Morocco. The rats were maintained on a 12 h light–dark cycle with a temperature of 22 °C ± 2 and provided with ad libitum access to standardized food and water. All animal procedures followed the guidelines outlined in the *Guide for the Care and Use of Laboratory Animals*, published by the United States National Research Council [31]. Additionally, the experiments were conducted in compliance with ethical principles and were approved by the Faculty of Sciences, Mohammed First University, Oujda, Morocco (Approval No. 7/24-LBBES).

### 2.4. Ex Vivo Assessment of the Vasorelaxant Effect of Crude Extracts

The vasorelaxant effect of crude extracts of *R. ulmifolius* was evaluated ex vivo using isolated aorta rings from normotensive male Wistar rats (*n* = 8) weighing between 200 and 300 g. The rats were anesthetized via intraperitoneal injection of sodium thiopental (0.1 mL/100 g body weight), and their thoracic aortas were excised, placed in a physiological solution, cleaned of adipose tissue, and cut into small rings measuring 2–3 mm in length. From each rat, three to four rings were obtained, and five rings were used per experimental condition. Each ring was suspended between two hooks and placed in an isolated organ chamber containing 11 mL of physiological solution composed of glucose (11 mM), magnesium sulfate (MgSO_4_, 1.2 mM), sodium chloride (NaCl, 119 mM), potassium dihydrogen phosphate (KH_2_PO_4_, 1.2 mM), potassium chloride (KCl, 4.7 mM), sodium bicarbonate (NaHCO_3_, 25 mM), and calcium chloride (CaCl_2_, 2.6 mM). The solution was continuously oxygenated (95% O_2_ and 5% CO_2_) and maintained at a pH of 7.4 and a temperature of 37 °C. The upper hook was connected to a force transducer (Emka technologies, France), and a tension of 1 g was applied to the mounted ring. The rings were allowed to stabilize for 30 min. After stabilization, the endothelial integrity of the aorta was assessed by inducing contraction with phenylephrine (PHE, 10^−6^ M) followed by relaxation with carbachol (CCh, 10^−4^ M). The experiment proceeded only if the relaxation exceeded 50%. Once endothelial integrity was confirmed, the rings were exposed to cumulative concentrations of each extract (10^−3^, 10^−2^, 10^−1^ g/L). No washing was performed between concentrations. Each subsequent concentration was added only after the vasorelaxant response to the previous concentration had reached a stable plateau, typically within 1 to 5 min. Methanol was used as the vehicle, and its effect was negligible. The percentage of relaxation was calculated as follows:

Vasodilation (%) = (Amplitude of relaxation/Amplitude of PHE contraction) × 100

### 2.5. In Vivo Assessment of the Hypotensive Effect of Methanolic Extract

The hypotensive effect was studied solely in the *R. ulmifolius* methanolic extract, identified as the most active in the vasorelaxant effect study. This investigation was conducted on normotensive male Wistar rats (n = 3) weighing between 220 and 360 g. The rats were anesthetized via intraperitoneal injection of sodium thiopental (0.1 mL/100 g body weight) and placed on a heating plate to maintain their body temperature at 37 °C. Cannulation was performed on the femoral vein and artery using pre-heparinized catheters to prevent blood coagulation. The catheter inserted into the femoral vein was used for administering the drug. In contrast, the one placed in the femoral artery enabled the recording and visualization of arterial signals using a National Instruments data acquisition system and LabVIEW 6.1 software. After a 30 min stabilization period, the baroreflex response was assessed by analyzing changes in blood pressure and heart rate following intravenous injection of phenylephrine (PHE, 3 µg/kg, an α-adrenergic agonist) and sodium nitroprusside (SNP, 10 µg/kg, an exogenous NO donor). Systolic blood pressure (SBP), diastolic blood pressure (DBP), mean arterial pressure (MAP), and heart rate (HR) were subsequently recorded before (control) and after intravenous administration of the methanolic extract at doses of 0.5, 1, 5, 10, and 20 mg/kg body weight, with a 10 min interval between each injection. The extract was administered by intravenous bolus injection at a volume of 25 µL/100 g of body weight. Each dose was individually adjusted according to the animal’s weight. Blood pressure variations were evaluated intra-individually by comparing the values recorded before and after each injection in the same rat. The vehicle used was 0.9% NaCl, and it had no effect.

### 2.6. Antioxidant Activity

The antioxidant activity of the methanolic extract of *R. ulmifolius* was evaluated in vitro using the DPPH assay with some modifications. A volume of 180 µL of a DPPH solution (150 µM in MeOH/H2O, 80:20) was added to 20 µL of each extract at varying concentrations (12.5, 25, 50, 100, 125, 150, and 250 µg/mL in methanol). The mixture was vigorously shaken and incubated in the dark at room temperature for 40 min. Trolox (12.5–250 µg/mL in methanol) was used as a standard. Absorbance was measured at 515 nm using a microplate reader. All experiments were performed in triplicate. The DPPH radical scavenging activity was calculated using the following equation:

% Radical scavenging activity = [1 − (As − Ab)/(Ac − Ab)] × 100

where Ab (blank absorbance) corresponds to the absorbance of MeOH/H_2_O (80:20) (180 µL) mixed with distilled water (20 µL); Ac (control absorbance) corresponds to the absorbance of the DPPH solution (180 µL) mixed with distilled water (20 µL); As (sample absorbance) corresponds to the absorbance of the DPPH solution (180 µL) mixed with the extract or Trolox (20 µL).

### 2.7. Quantitative Analysis of Total Phenolic and Tannin Content of Methanolic Extract

#### 2.7.1. Determination of Total Phenolic Content

The total phenolic content of the *R. ulmifolius* methanolic extract was determined using the Folin–Ciocalteu method, with some modifications. Briefly, 20 µL of the extract (0.42 mg/mL) was mixed with 100 µL of Folin–Ciocalteu reagent (diluted 1:10). The mixture was thoroughly shaken for 4 min. Subsequently, 80 µL of sodium carbonate (Na_2_CO_3_, 106 g/L) was added. The absorbance was measured at 760 nm. A standard curve was prepared using gallic acid at concentrations ranging from 0 to 100 mg/L. All measurements were performed in triplicate. The total phenolic content was expressed as milligrams of gallic acid equivalents per gram of extract (mg GAE/g).

#### 2.7.2. Determination of Total Tannin Content

The total tannin content of the *R. ulmifolius* methanolic extract was determined using the hide powder method, with some modifications. Initially, 1.5 mL of the extract (0.42 mg/mL) was heated in a water bath at 95 °C for 30 min. After heating, the extract was centrifuged for 5 min. Then, 500 µL of the supernatant was used to determine the total polyphenol content. An additional 500 µL of the supernatant was mixed with 10 mg of non-chromated hide powder (SIGMA^®^). The mixture was thoroughly stirred for one hour in the dark and then centrifuged for 5 min. The resulting supernatant was used to determine the total non-tannic polyphenol content (phenolic compounds not adsorbed by the non-chromated hide powder). The total tannin content was calculated as the difference between total polyphenols and non-tannic phenolic compounds. The results were expressed as milligrams of gallic acid equivalents per gram of extract (mg GAE/g).

### 2.8. Ultra-High-Performance Liquid Chromatography Coupled with Mass Spectrometry (UHPLC-MS) Analysis of Methanolic Extract

The separation of compounds from *R. ulmifolius* methanolic extract was performed using an Acquity UPLC H-Class Waters^®^ system (Guyancourt, France). The system comprised two independent pumps, a diode array detector (DAD), an automatic injector, a controller, and a QDa electrospray quadrupole mass spectrometer. Ionization was conducted in negative mode, with a mass range of 50–1250 Da. The cone voltage was set to 15 V, and the capillary voltage was maintained at 0.8 kV. The wavelength range for detection spanned 190–400 nm, with a resolution of 1.2 nm. The stationary phase consisted of an Uptisphere C18-AQ column (2.6 µm, 2.1 × 100 mm). The mobile phase was composed of two solvents: solvent (A) was ultrapure water with 0.1% formic acid (Carlo Erba Reagents^®^, Val de Reuil, France), and solvent (B) was acetonitrile (Carlo Erba Reagents^®^, Val de Reuil, France) with 0.1% formic acid. The elution gradient was programmed as follows: 5% to 80% (B) from 0 to 9 min, followed by 100% (B) from 9.5 to 10.5 min, and then a return to 5% (B) from 11 to 14 min. For the analysis, 1 mg of the extract was dissolved in 1 mL of methanol. The solution was filtered through a 20 µm Millipore filter, and 2 µL of the filtrate was injected. The analysis was carried out at 30 °C with a flow rate of 0.3 mL/min. The correspondence of each mass (m/z) to a molecule was essentially based on literature data. In addition, standards were used for some identified compounds [32,33,34]. Neochlorogenic acid, quercetin-3-O-β-D-glucuronide, kaempferol-3-O-β-D-glucuronide, and tiliroside were isolated from *R. ulmifolius* and characterized by NMR by our team. Chlorogenic acid, 3-*p*-coumaroylquinic acid, and ellagic acid were purchased from MedChemExpress (Nanterre, France) and Sigma-Aldrich (Saint-Quentin-Fallavier, France), respectively.

To quantify the different molecules identified in MERu, we used the same method as before, with some modifications. The wavelength ranges were 313 nm for 3-*p*-coumaroylquinic acid, 326 nm for chlorogenic acid, and 367 nm for ellagic acid. The elution gradient was programmed as follows: 5% (B) from 0 to 1 min, followed by 5% to 60% (B) from 1.01 to 9 min, 100% (B) from 9.01 to 11 min, and then a return to 5% (B) from 11.01 to 14 min.

### 2.9. Statistical Analysis

Results are expressed as mean ± SEM. Data were analyzed using a two-way ANOVA (Analysis of Variance), followed by Bonferroni’s post hoc test. All statistical analyses were performed using GraphPad Prism version 5 software. A *p*-value < 0.05 was considered statistically significant.

## 3. Results

### 3.1. Extraction Yield

The extraction yield was determined by calculating the ratio of the mass of the extract obtained after evaporation to the mass of the dried plant material. Among the extracts, the methanolic extract exhibited the highest yield. The yields of the hexane, dichloromethane, ethyl acetate, methanol, and aqueous extracts were 2.34%, 1.58%, 1.75%, 14.66%, and 11.62%, respectively.

### 3.2. Vasorelaxant Effect of the Crude Extracts

**Figure 1:**
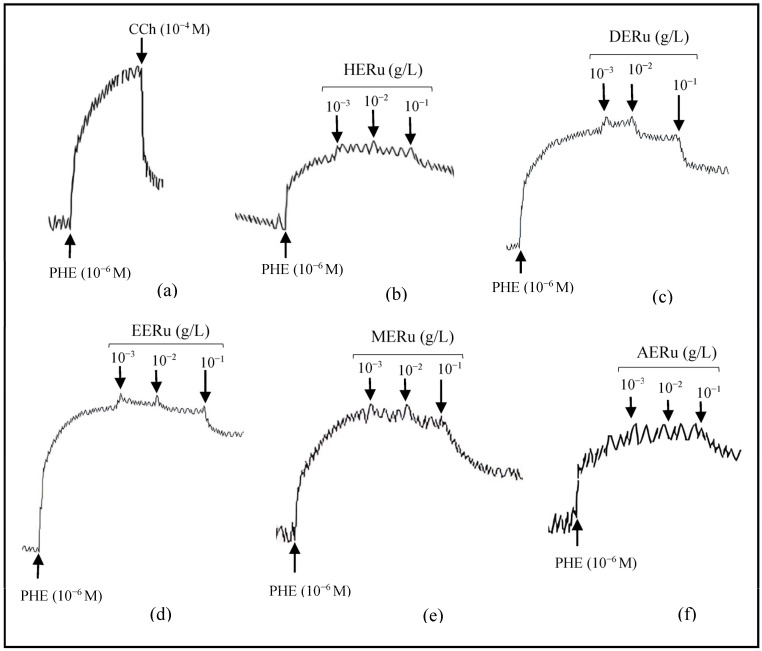
Original tracings demonstrating the vascular effects of carbachol (CCh, 10^−4^ M) (**a**) and cumulative doses (10^−3^, 10^−2^, 10^−1^ g/L) of crude extracts of *R. ulmifolius*: hexane (**b**), dichloromethane (**c**), ethyl acetate (**d**), methanol (**e**), and aqueous (**f**), on aorta precontracted with phenylephrine (PHE, 10^−6^ M).

**Figure 2:**
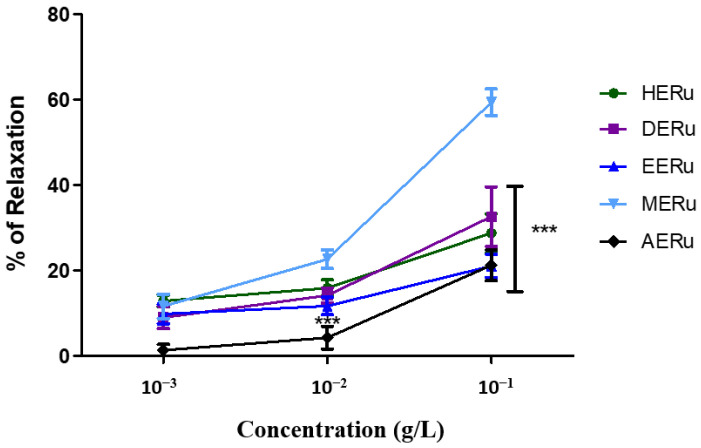
The vasorelaxant effect of cumulative doses (10^−3^, 10^−2^, 10^−1^ g/L) of the hexane (HERu), dichloromethane (DERu), ethyl acetate (EERu), methanol (MERu), and aqueous (AERu) extracts of *R. ulmifolius* on aorta precontracted with phenylephrine (PHE; 10^−6^ M). Results are expressed as mean ± standard error of the mean (SEM) (n = 5 rings, obtained from 5 rats per condition). *** *p* < 0.001 (MERu vs HERu, DERu, EERu, AERu), determined using two-way ANOVA followed by Bonferroni post hoc test.

Crude extracts of *R. ulmifolius* leaves—prepared using hexane (HERu), dichloromethane (DERu), ethyl acetate (EERu), methanol (MERu), and water (AERu)—induced concentration-dependent relaxation of aortic rings precontracted with phenylephrine. As shown in Figure 1 and Figure 2, the maximum vasorelaxation was observed at a concentration of 10^−1^ g/L, with the most pronounced effect exhibited by MERu, followed by DERu, HERu, AERu, and EERu. The percentages of maximum relaxation were 59.31 ± 3.09%, 32.57 ± 6.97%, 28.72 ± 4.49%, 21.22 ± 3.59%, and 21.03 ± 2.74%, respectively.

Since the methanolic extract (MERu) showed the best vasorelaxant effect, we decided to further investigate the biological effects of this extract.

### 3.3. Hypotensive Effect of the Methanolic Extract (MERu)

**Figure 3:**
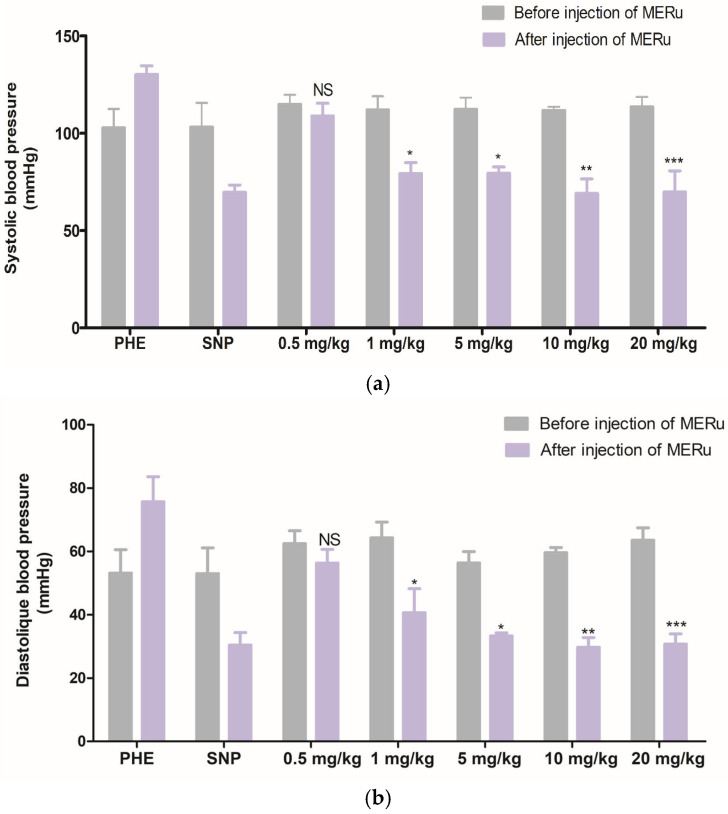
Effect of intravenous injection of the methanolic extract of *R. ulmifolius* (MERu) on systolic blood pressure (**a**) and diastolic blood pressure (**b**) in normotensive anesthetized rats. Results are expressed as mean ± SEM (n = 3 rats) and were analyzed using two-way ANOVA followed by Bonferroni post hoc test. NS (not significant), * *p* < 0.05, ** *p* < 0.01, *** *p* < 0.001 vs. control (before injection of the methanolic extract). PHE: phenylephrine; SNP: sodium nitroprusside.

The intravenous injection of the methanolic extract at doses of 0.5, 1, 5, 10, and 20 mg/kg body weight induced a decrease in systolic blood pressure (SBP) by 5.13 ± 1.68%, 29.24 ± 1.32%, 29.16 ± 3.18%, 38.24 ± 7.74%, and 38.61 ± 7.26%, respectively, and in diastolic blood pressure (DBP) 9.85 ± 4.46%, 36.74 ± 7.61%, 40.92 ± 4.25%, 50.11 ± 5.18%, and 51.58 ± 8.15%, respectively (Figure 3).

**Figure 4:**
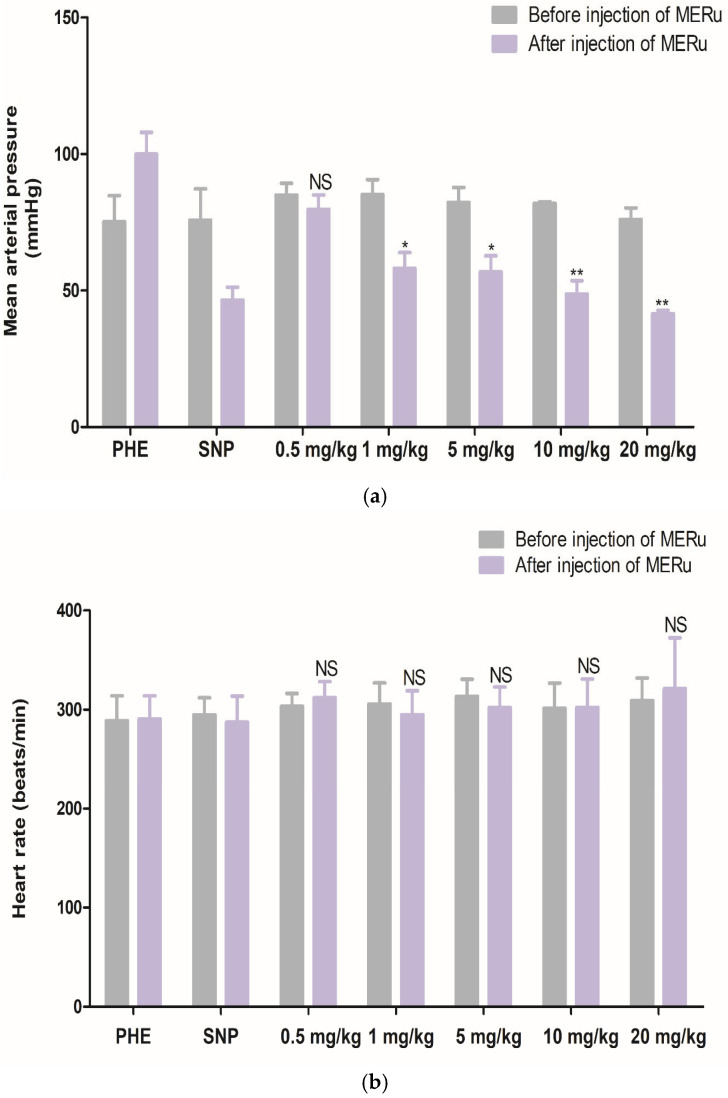
Effect of intravenous injection of the methanolic extract of *R. ulmifolius* (MERu) on mean arterial pressure (**a**) and heart rate (**b**) in normotensive anesthetized rats. Results are expressed as mean ± SEM (n = 3 rats) and were analyzed using two-way ANOVA followed by Bonferroni post hoc test. NS (not significant), * *p* < 0.05, ** *p* < 0.01 vs. control (before injection of the methanolic extract). PHE: phenylephrine; SNP: sodium nitroprusside.

For mean arterial pressure (MAP), reductions of 6.15 ± 2.83%, 31.75 ± 3.44%, 30.89 ± 5.28%, 39.96 ± 5.94%, and 45.19 ± 3.72% were observed at doses of 0.5, 1, 5, 10, and 20 mg/kg body weight, respectively. However, heart rate (HR) was not significantly affected by the methanolic extract (Figure 4).

### 3.4. Antioxidant Activity (DPPH Assay)

**Figure 5:**
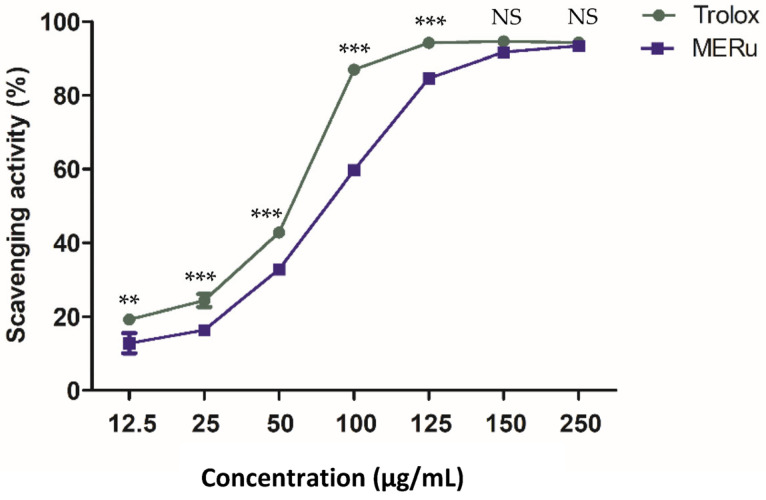
Antioxidant effect of the methanolic extract of *R. ulmifolius* (MERu) on DPPH radical scavenging (0.1 mM). Results are expressed as mean ± SEM (n = 3) and were analyzed using two-way ANOVA followed by Bonferroni post hoc test. NS (not significant), ** *p* < 0.01, *** *p* < 0.001 vs. Trolox.

The antioxidant effect of the methanolic extract of *R. ulmifolius* (MERu) was evaluated using the DPPH assay. The extract demonstrated a marked capacity to neutralize DPPH free radicals, reaching a maximum inhibition of 93.35 ± 0.39% at a concentration of 250 µg/mL, with an inhibitory concentration 50% (IC_50_) value of 86.49 µg/mL. As a reference, Trolox used as a positive control exhibited a maximum activity of 94.30 ± 0.23% at the same concentration, with an IC_50_ value of 59.18 µg/mL (Figure 5).

### 3.5. Total Phenolic, Tannin, and Pure Compound Contents of the Methanolic Extract

The phenolic and tannin contents of the methanolic extract of *R. ulmifolius* were determined using the Folin–Ciocalteu and the hide powder methods, respectively. The results are presented in Table 1. The extract exhibited phenolic and tannin contents of 218.36 ± 10.65 mg GAE/g and 139.58 ± 5.86 mg GAE/g, respectively.

Additionally, chlorogenic acid, 3-*p*-coumaroylquinic acid, and ellagic acid were quantified in MERu using standard ranges of their standard. However, neochlorogenic acid, quercetin-3-O-β-D-glucuronide, kaempferol-3-O-β-D-glucuronide, and tiliroside, isolated by our team, had a purity lower than 95%, so their quantification could not be performed. The different concentrations for the standard ranges of each standard, along with the evaluated parameters and the amount of each molecule in MERu, are presented in Table 1.

### 3.6. Ultra-High-Performance Liquid Chromatography Coupled with Mass Spectrometry (UHPLC-MS) Analysis of the Methanolic Extract

**Figure 6:**
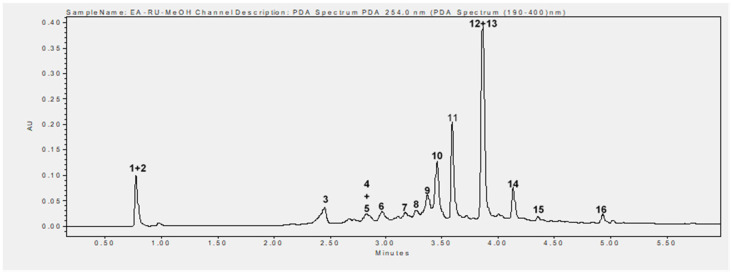
UHPLC chromatogram recorded at 254 nm. 1. Not identified, 2. rubanthrone A, 3. neochlorogenic acid, 4. *p*-coumaroylquinic acid derivative, 5. kaempferol-3-O-arabinoside, 6. chlorogenic acid, 7. kaempferin, 8. epicatechin, 9. 3-*p*-coumaroylquinic acid, 10. galloyl-bis-HHDP glucose derivative, 11. not identified, 12. ellagic acid, 13. quercetin-3-O-β-D-glucuronide, 14. kaempferol-3-O-β-D-glucuronide, 15. not identified, 16. tiliroside.

Phytochemical analysis of the methanolic extract using UHPLC-MS identified 16 phenolic compounds, including flavonoids, tannins, and phenolic acids. The main compounds identified were rubanthrone A (peak 2), a galloyl-bis-HHDP glucose derivative (peak 10), ellagic acid (peak 12), and quercetin-3-O-β-D-glucuronide (peak 13) (Table 2, Figure 6). These compounds were identified by comparing their mass spectral data and retention times with those reported in the literature [32,34].

Additionally, the retention times of neochlorogenic acid, chlorogenic acid, 3-*p*-coumaroylquinic acid, ellagic acid, quercetin-3-O-β-D-glucuronide, kaempferol-3-O-β-D-glucuronide, and tiliroside were compared with those of their respective standards.

## 4. Discussion

In our previous study, a nutrigenomic experiment was conducted using mouse models with *R. ulmifolius* Schott (wild blackberry) and *Artemisia campestris* L. (mugwort) leaves. We only focused on the aqueous extracts prepared by infusion, with the aim of associating biomarker genes with the vasorelaxant and hypotensive effects of these extracts [25]. Despite the promising cardiovascular effects observed from the aqueous extracts, this approach did not allow for the precise identification of the active compounds, nor did it reveal whether more potent molecules, not extracted by water, might be present. This encouraged us to continue investigating *Rubus ulmifolius*. Thus, the current study aimed to investigate the vasorelaxant effect of different crude extracts of *R. ulmifolius* (HERu, DERu, EERu, MERu, AERu), using solvents of increasing polarity, on the isolated rat aorta precontracted with phenylephrine (10^−6^ M). Based on the results obtained, the addition of cumulative concentrations (10^−3^, 10^−2^, 10^−1^ g/L) of the different extracts induced vasorelaxation, with the maximal effect observed at a concentration of 10^−1^ g/L. The methanolic extract (MERu) exhibited the most potent effect, with a maximum relaxation of 59.31%.

To understand through which pathway this effect occurs, further mechanistic investigations, particularly using nitric oxide synthase inhibitors such as L-NAME, would be useful to determine whether the observed vasorelaxant effect is endothelium-dependent.

Vasodilation (dilation of blood vessels) contributes to maintaining normal blood pressure by reducing vascular resistance (resistance to blood flow). It is mediated by the endothelium, the innermost layer of blood vessels, which plays a central role in regulating vascular homeostasis [35]. The endothelium is responsible for secreting vasodilatory substances such as nitric oxide (NO), endothelium-derived hyperpolarizing factors (EDHFs), and prostacyclin (PGI_2_). Under pathological conditions, blood vessels become rigid, and the endothelium loses its functions, leading to a reduction in the secretion of these substances. This promotes vasoconstriction and, consequently, the development of hypertension [36].

The vasorelaxant effect of MERu, reaching 59.31% at a concentration of 10^−1^ g/L, is comparable to that of the methanolic extract of *Tetraclinis articulata*, obtained by Soxhlet extraction, which induced a relaxation of 56% at the same concentration in isolated rat aortic rings [37]. Similarly, a vasorelaxant effect of the methanolic extract of *Chenopodium ambrosioides*, also obtained by Soxhlet extraction and tested on isolated rat aortic rings, was observed at various concentrations (10^−3^, 10^−2^, 10^−1^, and 1 mg/mL), with a maximal relaxation of 35.5% at 10^−1^ mg/mL [38]. Thus, at an equivalent concentration, the vasorelaxant effect of MERu appears more pronounced than that of the methanolic extract of *C. ambrosioides*.

Due to its potent vasorelaxant effect, MERu was selected to investigate its hypotensive effect in anesthetized normotensive rats. Our results showed that intravenous injection of MERu at increasing doses (0.5, 1, 5, 10, 20 mg/kg) induced a significant reduction in blood pressure starting at the dose of 1 mg/kg of body weight. At the dose of 20 mg/kg of body weight, the extract reduced systolic blood pressure, diastolic blood pressure, and mean arterial pressure by 38.61%, 50.33%, and 45.33%, respectively. However, the heart rate remained unchanged. This suggests that the hypotensive effect of MERu is mediated through the vascular pathway and is independent of the cardiac pathway.

Previous studies on other berry species have highlighted beneficial effects on vascular relaxation and blood pressure reduction. Anthocyanin-rich extracts of *Aronia melanocarpa* (chokeberry) and *Vaccinium myrtillus* (bilberry) induced concentration-dependent relaxation in isolated porcine coronary artery rings, achieving maximal effects of 68% and 59%, respectively, at a concentration of 5 mg/L [39]. In another study, an *Aronia melanocarpa* extract reduced blood pressure by 21% in L-NAME-treated hypertensive rats [40]. It has also been suggested that blueberry supplementation in rats fed a high-fat, high-cholesterol diet improved relaxation in isolated aortic rings in response to acetylcholine and significantly reduced systolic blood pressure [41]. Additionally, the ethanol extract of *Rubus coreanus* Miq. (black raspberry) fruits induced concentration- and endothelium-dependent relaxation in isolated rat aorta precontracted with phenylephrine, with a maximal effect of 93.84% at a concentration of 10 μg/mL [42]. Furthermore, the aqueous and butanol fractions of *Crataegus songarica* (hawthorn berry) demonstrated endothelium-dependent vasorelaxant effects, with the aqueous fraction being the most active, inducing 94% relaxation in isolated porcine coronary artery rings at a concentration of 3 mg/mL. In contrast, the butanol fraction caused 73.5% vasorelaxation at a concentration of 10 mg/mL [43].

Another part of this work focused on studying the antioxidant activity of MERu using the DPPH assay. Oxidative stress plays a key role in the development of hypertension. It results from an imbalance between the formation of reactive oxygen species (ROS) and their neutralization by antioxidant systems. Among the main sources of ROS production in blood vessels is the NADPH oxidase enzyme, which contributes to the inactivation of NO. It has been demonstrated that isolated arteries from hypertensive animals or humans exhibited a significant increase in ROS production [44]. The results of the DPPH assay showed that MERu possessed a potent antioxidant effect (Figure 5).

Previous studies on *R. ulmifolius* have also revealed antioxidant activity through this test. Chloroform, methanolic, and aqueous extracts demonstrated antioxidant activity with respective IC_50_ values of 45.54 μg/mL, 2.13 μg/mL, and 4.13 μg/mL [45]. Additionally, it was observed that the methanolic extract exhibited a potent antioxidant effect, with an IC_50_ value of 5.10 μg/mL [46]. Antioxidants are molecules capable of neutralizing ROS and act through various mechanisms. Among them, polyphenols represent an important class of antioxidants. They have shown beneficial effects on endothelial function and blood pressure regulation, particularly by inhibiting NADPH oxidase and increasing the expression of the endothelial nitric oxide synthase (eNOS), the enzyme responsible for NO synthesis [44].

Our study revealed that MERu contained high levels of polyphenols such as tannins (Table 1). To identify the specific compounds, present in this extract, a phytochemical analysis was conducted using ultra-high-performance liquid chromatography coupled with mass spectrometry (UHPLC-MS). This analysis showed that MERu is primarily composed of phenolic acids (neochlorogenic acid, *p*-coumaroylquinic acid derivative, chlorogenic acid, 3-*p*-coumaroylquinic acid), tannins (epicatechin, ellagic acid, galloyl-bis-HHDP glucose derivative), and flavonoids (kaempferol-3-O-arabinoside, kaempferin, quercetin-3-O-β-D-glucuronide, kaempferol-3-O-β-D-glucuronide, tiliroside).

Flavonoids are diverse polyphenolic compounds known for their beneficial effects on the cardiovascular system, particularly due to their ability to reduce the risk of hypertension and other cardiovascular diseases. Quercetin and kaempferol are flavonols that can exist in glycosylated forms. It has been demonstrated that quercetin lowers blood pressure through several mechanisms, including improving endothelial function, exerting antioxidant activity, and inhibiting angiotensin-converting enzyme (ACE) [47]. Additionally, a study revealed that kaempferol reduced blood pressure and induced relaxation of isolated aortic rings from rats precontracted with phenylephrine by stimulating endothelial NO production [48]. Moreover, it was reported that tiliroside reduced blood pressure in DOCA-salt hypertensive rats and dilated mesenteric arteries precontracted with phenylephrine. This vasorelaxant effect was endothelium-independent and occurred through the blockade of L-type calcium channels in smooth muscle cells [49]. Furthermore, a molecular docking study revealed that pentose ellagic acid, tiliroside, and galloyl-HHDP glucose had binding affinity for ACE, which is responsible for converting angiotensin I to angiotensin II, a potent vasoconstrictor. This affinity for ACE suggests an inhibition of its activity [50]. Additionally, research has shown that epicatechin reduced blood pressure and preserved cardiac function in DOCA-salt hypertensive rats with cardiac hypertrophy [51].

Regarding phenolic acids, chlorogenic acid is recognized for its health benefits, particularly its antihypertensive properties. A study demonstrated that it induced concentration and endothelium-dependent relaxation in phenylephrine-precontracted rat aortic rings, with a maximal effect of 67% at a concentration of 10^−4^ M. This effect was mediated through the activation of signaling pathways involving EDHF, eNOS, and cyclooxygenase [52]. Furthermore, a randomized study conducted on healthy individuals demonstrated that chlorogenic acid significantly reduced systolic and diastolic blood pressure [53].

Based on these results, chlorogenic acid, epicatechin, quercetin-3-O-β-D-glucuronide, kaempferol-3-O-β-D-glucuronide, and tiliroside, identified in the methanolic extract of *R. ulmifolius*, may play a key role in the observed vasorelaxant and hypotensive effects.

However, it is well established that the in vivo efficacy of phytochemicals largely depends on their bioavailability and pharmacokinetic profile. Taking these parameters into account is therefore essential to accurately assess the extent of their systemic effects. Among the compounds identified in the methanolic extract of *R. ulmifolius*, ellagic acid, galloyl-bis-HHDP glucose derivatives, and quercetin-3-O-β-D-glucuronide are particularly noteworthy, as they are major constituents of the extract and have been previously investigated for their pharmacokinetic behavior.

Ellagic acid is known for its low oral bioavailability, primarily due to poor absorption and limited solubility. A study conducted in rats by Lei et al. (2003) showed that following oral administration of a pomegranate leaf extract (0.8 g/kg) rich in ellagic acid, the compound reached a maximum plasma concentration of 213 ng/mL at 33 min post-ingestion, but was rapidly eliminated [54]. In humans, Seeram et al. (2004) demonstrated that after ingestion of pomegranate juice containing 25 mg of ellagic acid, the compound was detected in plasma one hour after administration at a peak concentration of only 31.9 ng/mL and became undetectable after four hours [55]. González-Sarrías et al. (2015) reported similar findings even after higher doses, highlighting extensive microbial metabolism into urolithins [56].

Regarding galloyl-bis-HHDP glucose derivatives, these compounds belong to the ellagitannin class. A study conducted in Iberian pigs, a physiological model closely related to humans, showed that after consumption of an acorn-rich diet containing ellagitannins, these compounds were no longer detectable in plasma. They were first hydrolyzed into ellagic acid, which was then metabolized by the gut microbiota into urolithins. These more lipophilic metabolites are better absorbed and represent the predominant forms detected in systemic circulation [57]. Similarly, a study in human volunteers demonstrated that after consumption of red raspberries rich in ellagitannins, the parent compounds were not detected in plasma. However, their metabolites, mainly conjugated urolithins, were present. This suggests that this metabolic transformation is essential to account for the systemic effects of ellagitannins despite their poor absorption in native form [58]. Additionally, a recent pharmacokinetic study in rabbits on geraniin, another ellagitannin, revealed that it is not absorbed as such but is hydrolyzed in the digestive tract into ellagic acid, which itself is poorly absorbed. However, the authors developed an optimized formulation, a geraniin–phosphatidylcholine complex, to improve the solubility and lipophilicity of ellagic acid and thereby enhance its absorption. With this formulation, ellagic acid was detected in plasma as early as 0.5 h post-administration, with two concentration peaks (Cmax) of 588.82 ng/mL at 2 h (Tmax_1_) and 711.13 ng/mL at 24 h (Tmax_2_), reflecting a marked improvement in bioavailability [59].

As for quercetin-3-O-β-D-glucuronide, a comparative pharmacokinetic study in rats evaluated the profiles of quercetin, isoquercitrin, and quercetin-3-O-β-D-glucuronide following oral administration of each compound at a dose of 50 mg/kg. All three compounds were detected in plasma, confirming systemic absorption. Moreover, following administration of quercetin-3-O-β-D-glucuronide, free quercetin was also detected in plasma as early as 1.5 h, indicating in vivo metabolic conversion. The bioavailability of quercetin, estimated from the area under the plasma concentration–time curve (AUC_0_–t), was 3505.7 ± 1565.0 mg × min/L, while that of quercetin-3-O-β-D-glucuronide was 962.7 ± 602.3 mg × min/L, suggesting that part of the latter is converted into quercetin, the more bioavailable form [60].

These pharmacokinetic limitations considerably reduce the systemic efficacy of these compounds when administered orally. In our study, intravenous administration of the methanolic extract of *R. ulmifolius* allowed the active constituents to reach the systemic circulation directly, thereby bypassing first-pass metabolism and intestinal absorption barriers, which may explain the cardiovascular effects observed.

These findings provide important insights into the pharmacological potential of *R. ulmifolius* and support its relevance in the management of cardiovascular disorders. Further investigations into the pharmacokinetics of isolated compounds from *R. ulmifolius*, as well as into their long-term cardiovascular effects, would be valuable to confirm and extend these findings.

## 5. Conclusions

The findings of this study demonstrated the concentration-dependent vasorelaxant effect of crude extracts of *R. ulmifolius* (hexane, dichloromethane, ethyl acetate, methanol, and aqueous) on isolated rat aortic rings precontracted with phenylephrine, with the most pronounced effect induced by the methanolic extract (MERu). Furthermore, intravenous administration of MERu in anesthetized normotensive rats resulted in a significant reduction in blood pressure starting at a dose of 1 mg/kg body weight. The extract also exhibited notable antioxidant activity. Phytochemical analysis of MERu, conducted using UHPLC-MS, confirmed the presence of flavonoids, phenolic acids, and tannins, suggesting that these compounds may contribute to the observed effects. These findings highlight the significant vasodilatory and hypotensive effects of the MERu of *R. ulmifolius*.

*Rubus ulmifolius* shows promising characteristics as a potential functional food, owing to its rich phytochemical content and demonstrated antioxidant, vasorelaxant, and hypotensive activities. These results align with the fact that no toxicity has been reported in the leaves, supporting their use in food.

Further studies are needed to isolate the major bioactive compounds, evaluate their effects in pure form, elucidate the underlying mechanisms of action, and validate the extract’s potential role in cardiovascular health through human trials.

## Figures and Tables

**Table 1:** Total phenolic, tannin, and pure compound contents of *R. ulmifolius* methanolic extract.

Content *	Concentration Range (µg/mL)	Number of Points	R^2^	Result
Total phenolic	-	3	1	218.36 ± 10.67
Total non-tannic phenolic	-	3	1	78.78 ± 4.81
Total tannin	-	3	1	139.58 ± 5.86
Chlorogenic acid	10–100	6	0.991	4.33 ± 1.52 < LOQ
3-*p*-Coumaroylquinic acid	10–100	6	0.997	1.0 ± 0.0 < LOQ
Ellagic acid	10–100	6	0.999	20.0 ± 0.0

Note: Values in the table are expressed as means ± SEM (n = 3), MERu: methanolic extract, LOQ: limit of quantification. * Total phenolic, non-tannic phenolic, and total tannin contents are expressed as mg GAE/g, while pure compounds are expressed as μg/mg.

**Table 2:** UHPLC-MS analysis of MERu.

Peak	Retention Time (min)	Molecular Ion [M-H]-(*m*/*z*)	Tentative Identification
1	0.740	215.20	NI
2	0.740	377.24	Rubanthrone A
3	2.450	353.22	Neochlorogenic acid
4	2.824	337.18	*p*-Coumaroylquinic acid derivative
5	2.824	417.32	Kaempferol-3-O-arabinoside
6	2.966	353.27	Chlorogenic acid
7	3.170	431.41	Kaempferin
8	3.267	289.14	Epicatechin
9	3.367	337.21	3-*p*-Coumaroylquinic acid
10	3.458	934.39	Galloyl-bis-HHDP glucose derivative
11	3.587	433.26	NI
12	3.862	301.09	Ellagic acid
13	3.862	477.29	Quercetin-3-O-β-D-glucuronide
14	4.140	461.27	Kaempferol-3-O-β-D-glucuronide
15	4.369	547.38	NI
16	4.942	593.34	Tiliroside

Abbreviations. NI: not identified.

## Data Availability

All data supporting the results of this study are presented in the manuscript.

## Abbreviations

The following abbreviations are used in this manuscript:

MERu	Methanolic extract of *Rubus ulmifolius*
HERu	Hexanic extract of *Rubus ulmifolius*
DERu	Dichloromethane extract of *Rubus ulmifolius*
EERu	Ethyl acetate extract of *Rubus ulmifolius*
AERu	Aqueous extract of *Rubus ulmifolius*
PHE	Phenylephrine
CCh	Carbachol
SNP	Sodium nitroprusside
SBP	Systolic blood pressure
DBP	Diastolic blood pressure
MAP	Mean arterial pressure
HR	Heart rate
